# Energy Harvesting Technologies for Achieving Self-Powered Wireless Sensor Networks in Machine Condition Monitoring: A Review

**DOI:** 10.3390/s18124113

**Published:** 2018-11-23

**Authors:** Xiaoli Tang, Xianghong Wang, Robert Cattley, Fengshou Gu, Andrew D. Ball

**Affiliations:** 1 Centre for Efficiency and Performance Engineering, School of Computing and Engineering, University of Huddersfield, Huddersfield HD1 3DH, UK; Xiaoli.Tang@hud.ac.uk (X.T.); R.Cattley@hud.ac.uk (R.C.); F.Gu@hud.ac.uk (F.G.); A.Ball@hud.ac.uk (A.D.B.); 2 School of Automobile and Mechanical Engineering, Changsha University of Science and Technology, Changsha 410004, China

**Keywords:** energy harvesting systems, machine condition monitoring, wireless sensor networks, maintenance-free

## Abstract

Condition monitoring can reduce machine breakdown losses, increase productivity and operation safety, and therefore deliver significant benefits to many industries. The emergence of wireless sensor networks (WSNs) with smart processing ability play an ever-growing role in online condition monitoring of machines. WSNs are cost-effective networking systems for machine condition monitoring. It avoids cable usage and eases system deployment in industry, which leads to significant savings. Powering the nodes is one of the major challenges for a true WSN system, especially when positioned at inaccessible or dangerous locations and in harsh environments. Promising energy harvesting technologies have attracted the attention of engineers because they convert microwatt or milliwatt level power from the environment to implement maintenance-free machine condition monitoring systems with WSNs. The motivation of this review is to investigate the energy sources, stimulate the application of energy harvesting based WSNs, and evaluate the improvement of energy harvesting systems for mechanical condition monitoring. This paper overviews the principles of a number of energy harvesting technologies applicable to industrial machines by investigating the power consumption of WSNs and the potential energy sources in mechanical systems. Many models or prototypes with different features are reviewed, especially in the mechanical field. Energy harvesting technologies are evaluated for further development according to the comparison of their advantages and disadvantages. Finally, a discussion of the challenges and potential future research of energy harvesting systems powering WSNs for machine condition monitoring is made.

## 1. Introduction

Condition monitoring is a process of judging the health status of a mechanical system, which uses various types of data (such as temperature, vibration, strain, rotating speed, displacement, pressure, voltage, current, acoustics and operator experience) to achieve change-point detection and thus provide a timely decision for the maintenance works [1]. Machine condition monitoring delivers significant benefits of cost savings, safety and reliability to industries by providing an early indication of potential machine failure in the machine operation cycle. As such, condition monitoring has attracted substantial attention from companies and research institutions for decades [2,3,4]. Traditionally, plenty of wires or cables are required in a condition monitoring system to transfer data from various transducers to data acquisition devices. High costs and difficult installations, along with low operational reliability are often the main drawbacks of using these wired systems. To overcome such drawbacks, latest wireless sensor networks (WSNs) have become an effective and efficient solution. In addition to providing key advantages of low-cost installation and operation, WSN also has the merits of low power consumption, high flexibility and distributed intelligence in implementing remote real-time condition monitoring.

**Figure 1:**
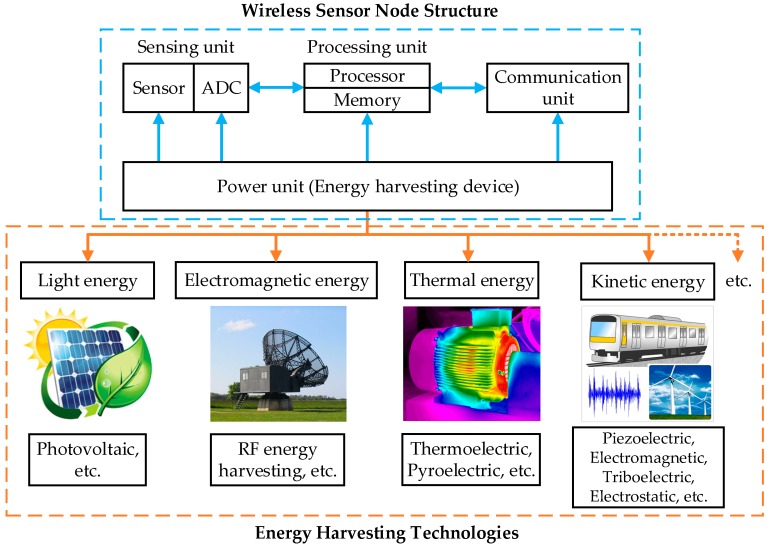
Wireless sensor nodes powered with energy harvesting techniques.

Generally, a wireless sensor node in WSN is composed of four key units [5]: a sensing unit, a processing unit, a communication unit and a power unit as shown in Figure 1. The power unit poses a significant problem because conventional batteries have a finite lifespan, limited energy density and capacity. In addition, the performance of batteries has not improved much compared to the significant increase of the power consumption in electronic devices [6]. When the batteries are exhausted, replacing or recharging it can be an expensive and difficult task especially when the nodes are remote or inaccessible. Fortunately, the wasted energy from machines or its surrounding environments, such as thermal energy, magnetic and electric fields and mechanical energy, can be harvested to power the sensor nodes by means of energy harvesting (EH) technologies [7], which is the procedure of converting wasted energy from ambient sources into electrical energy [8]. This approach substantially prolongs the life of sensing nodes; furthermore, it reduces maintenance costs of the monitoring system and avoids the environmental contamination of batteries.

As shown in Figure 1, wasted energy sources (such as light, electromagnetic radiation, heat, vibration, motion and magnetic energy) can be harvested using various traditional EH techniques (usually including photovoltaic [9], radio frequency (RF), thermoelectric, pyroelectric [10], piezoelectric, electromagnetic, triboelectric and electrostatic [11,12]). Currently, these EH techniques are primarily targeted at small and ultra-low power devices, like portable electronic devices, wearable devices and WSNs [13].

For mechanical systems, the energy losses are present in power transformation and transmission in the form of friction, heat, deformation and vibration during operation. Therefore, it is recognized that mechanical efficiency is always below 100%. Besides, more efficient, renewable and generally inexhaustible energy sources are likely to exist in the environment around the mechanical systems. These different forms of energy provide the possibility of supplementing or replacing additional batteries for supplying power to WSNs for machine condition monitoring in order to achieve a true wireless and maintenance-free system. In the last decade, innumerable researchers have contributed to the technology of energy extraction from machine systems [14,15,16,17,18,19]. Though significant progress has been made in various aspects, these EH technologies still have challenging deficiency of providing insufficient electricity to power the sensor nodes of WSN for real-time machine condition monitoring.

Numerous algorithms relating to the energy-efficient routing of WSNs have been proposed recently, such as effective node-selection schemes [20], distributed routing schemes for energy management [21]. Sherazi et al. [22] makes a comprehensive survey of EH and discussed the challenges and trade-offs of media access control (MAC) protocols in WSNs. Then, devices designed with innovative structures or novel materials have been studied to enhance energy conversion efficiency [23,24,25]. Furthermore, because large-scale EH devices are affected by the area available for installation on target objects, designers must face the challenges of small size and high conversion efficiency on the EH devices acting at microwatt levels. Therefore, nanotechnology has attracted researchers’ attention on the design and fabrication of energy harvesting nanoscale devices. Chen et al. [26] summarizes the triboelectric nanogenerators proposed in the past few years. Most of them work for low-frequency actions, such as human activities, rotating machines and bridge structures. The state-of-the-art nanotechnology and fabrication technology create opportunities for a combination of EH technologies. As a result, hybrid nanogenerators have improved the performance of energy harvesters. He et al. [27] has fabricated a hybrid nanogenerator through integrating the triboelectric and piezoelectric effects with electromagnetic induction to supply continuous and reliable power for transmission of temperature and vibration data.

With the rapid advancement in new materials and nanotechnologies along with more efficient WSN topologies and constructions, more and more EH devices have been successfully developed and applied in various fields, like human health monitoring, portable electronic devices, wearable devices, as the shown in surveys made by [6,7,28]. These EH based self-powered WSNs have the potential for producing an economical, flexible, efficient, reliable and accurate condition monitoring system due to the development of EH technologies and WSNs. Unfortunately, such EH apparatuses have been found to be infrequently used for the long-term and real-time machine condition monitoring. In order to encourage the extensive and prevalent applications of EH in maintenance-free machine condition monitoring, a comprehensive literature review is essential to prompt the application of energy generators to achieve high performance and maintenance-free wireless sensor nodes for machine condition monitoring. To this end, this paper summarizes the physical principles of various EH technologies, enumerates their applications and discusses their potential prevalent applications in the field of machine condition monitoring. To the authors’ knowledge, this is the first review on self-powered WSNs based on energy harvesting applied for machine condition monitoring.

In this review paper, conventional wireless communication technologies are first introduced and the power consumption of WSNs are discussed. Then, energy sources from machinery systems are analysed and evaluated for future harvesting usage. Next, the theory of traditional EH technologies and their improvements are introduced. The wide applications of these EH technologies and prototypes are surveyed in a variety of fields, especially in the machinery domain. Simultaneously, the advantages and disadvantages of various technologies and models are summarized. Lastly, the challenges and future developments of EH technologies applied to machine condition monitoring is investigated and recommendations are made.

## 2. Power Demands and Resources of WSN based Condition Monitoring

As shown in Figure 1 electric energy in a WSN primarily supplies power the communication unit, processing unit and sensing unit. Moreover, the power consumption of WSNs largely depends upon applications which define the actual transmission rate and distance required to complete the tasks of an application. 

### 2.1. Power Consumption of a WSN based System

Wireless communication technology has developed rapidly with domestic and industrial demands from the end of the last century, developing many different WSNs. The standardized wireless transmission technologies mainly comprise Wi-Fi, Bluetooth low energy (BLE), ZigBee and high-frequency RF (such as 433 MHz, 915 MHz, 2.45 GHz and 5.8 GHz) [29]. EnOcean, Z-wave and ANT, as representatives of emerging proprietary communication technologies, have shown potential developments in smart homes, automation control, transportation and logistics, though these systems have not yet dominated the market [30]. Nevertheless, these low power consumption WSNs, as reviewed in Table 1, provide the fundamentals to realise a self-power system through effective EH from its surroundings, for example from motion, light or temperature differences.

Currently, there are a number of popular modules available for developing WSN applications. As summarized in Table 2, these modules are fabricated based on the wireless communication technologies in Table 1. However, due to differences in applications and advances in fabrication technologies used, they exhibit different performances of energy usages even if they are from the same communication technology. It shows that the energy consumed is generally at the level of milliwatts or watts. Both Wi-Fi and BLE are the most energy efficient transmission methods by evaluation of data rate and power consumption, which have the potential to be competitors of other wireless communication technologies for the achievement of remote and real-time machine condition monitoring taking into account of their relatively long transmission distance and high transmission rate.

The power consumed by processing and sensing units is used for data collection and data processing. Table 3 enumerates typical microprocessor modules with active consumption at about watt level. In reality, the electricity cost by a microprocessor is dependent up on the instructions processed. In other words, the implementation of massive complex algorithms results in a significant increase in power consumption of a sensor node microprocessor, for example, from dozens of milliwatts or hundreds of milliwatts. 

In contrast, the sensing unit consumes the least power, generally at the microwatt or milliwatt level, which can be certified from the parameters of sensor modules as shown in Table 4. It is also deduced that the transmission unit of a wireless sensor node consumes the most energy causing power consumption of an entire node to be at the milliwatt to watt level, the comparison of various modules are shown in Table 2, Table 3 and Table 4.

The main physical parameters measured for machine condition monitoring include temperature, humidity, torque, stress, strain, pressure, speed, vibration and acoustic emission. The lower varying signals, such as temperature are suggested for a network with a low transmission rate and power consumption, typically ZigBee, EnOcean, Z-wave and ANT. However, it is difficult to determine the early fault diagnosis and fault location using such limited data. The vibrational characteristics of the mechanical faults are usually found at a much higher frequency band, which requires a high sampling frequency according to the Nyquist-Shannon sampling theorem, resulting in a large number of raw datasets for processing and transmission. Although feature extraction at nodes consumes less power than the direct transmission of raw datasets, some advanced algorithms for extracting fault features with high computational costs, for example, for example Spectral Correlation [37] and Modulation Signal Bispectrum [38,39], cannot be operated at nodes. Therefore, it is still essential to transmit large numbers of raw datasets through a WSN with a high transmission speed to implement the remote and real-time machine condition monitoring. The relationship between power consumption and transmission rate has been researched, unveiling that the long-term data transmission at a low rate can consume more power than a short-term transmission at a high rate when the effect of transmission distance is excluded [40,41]. Therefore, a wireless network with a higher transmission rate (for example BLE, Wi-Fi and ZigBee) is critical in order to achieve remote machine condition monitoring in real time. BLE 4.2 consumes less power, but the actual transmission distance is limited to 10 m. It is attractive that BLE 5.0, a new generation of Bluetooth, has a wider coverage range and a higher speed compared to BLE 4.2 for low-power devices. This provides an opportunity to achieve a large amount of datasets transmission in real time with relatively low power consumption in online machine condition monitoring.

The improvement of intelligent networks and big data analytics provides another solution for self-powered machine condition monitoring systems. MCUs, such as the ARM Cortex-M series, have vast processing capacity with the power consumption levels for data processing being substantially less than the power used for data transmission. As a result, a large amount of sampled data can be partially pre-processed in the MCU, and then the signal features are extracted and transmitted instead of transmitting the full raw data which provides significant power saving. In general, a trade-off between the network selection and energy harvesting should be considered before a wireless condition monitoring system is implemented. Moreover, there should be a balance between data processing and data transmission in algorithm design and network optimization. In general, power at the milliwatt level or watt level is required to process and transmit large amounts of rapidly varying signals at a wireless sensor node for remote and real-time machine condition monitoring.

### 2.2. Potential Energy Harvesting Sources in Machines

**Figure 2:**
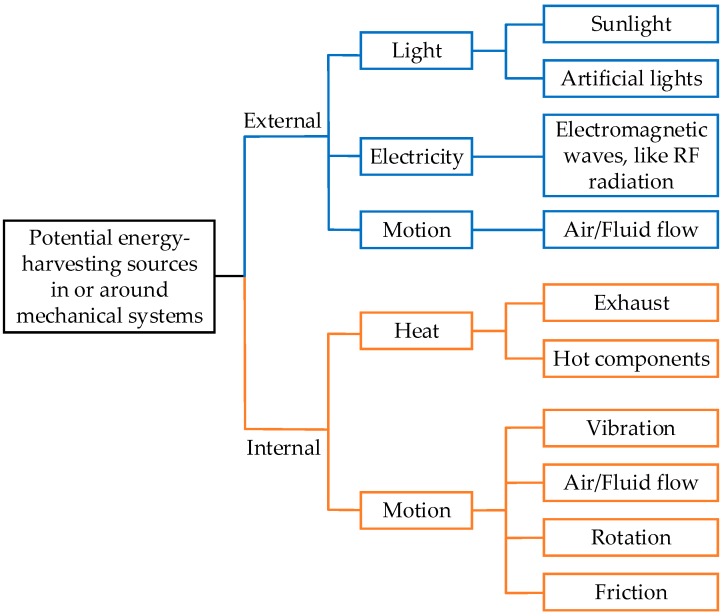
Potential energy-harvesting sources in mechanical systems.

To provide sufficient power for self-powered WSNs, the potential energy harvesting resources in or around mechanical systems can be analyzed as shown in Figure 2. In general, these sources can be split into internal and external energy sources representing the energy coming from mechanical systems or their surroundings. Then, they are further classified into light, electricity, heat and motion based on the forms of energy.

Solar energy is a green, renewable and efficient source in the environment where most mechanical systems are located. Solar radiation at a high level can be harvested and stored. However, sunlight is crucially affected by the region, weather and length of day. Artificial light has little correlation with these factors, but its illumination level is several orders of magnitude lower than that of sunlight. Electromagnetic waves, such as RF radiation, are another energy source mainly existing in the surroundings due to the wide usage of radio transmitters. Both light and RF radiation energy can be harvested to use for wireless body area networks [36,42]. The force generated by fluid flows (like wind and tidal current [43]) is an effective source of kinetic energy. For example, marine tidal energy [44] can be harvested for large-scale equipment on the seashore or ships. External energy sources are greatly influenced by the environment, and the corresponding generators may be difficult to adapt to condition monitoring systems. Their common applications are power generation for the power plants [45,46,47].

**Figure 3:**
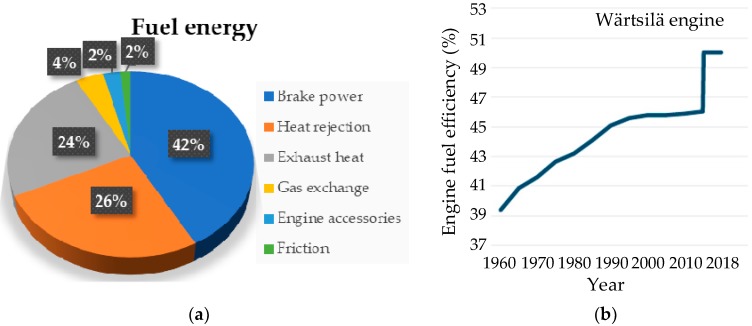
Energy efficiency of engines: (**a**) a typical diesel engine in 2009; (**b**) Wärtsilä engine fuel efficiency development.

**Figure 4:**
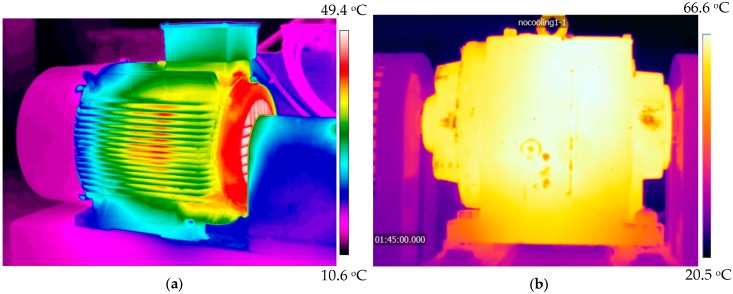
Local thermal imaging of mechanical components: (**a**) Motor; (**b**) Gearbox housing.

In contrast, the internal energy of machines is relatively reliable and steady. Efficiency of the power machines (usually IC engines and motors) is less than 50%. The wasted energy gives a chance to be harvested and then used to power the WSN for monitoring the working conditions. For example, the efficiency of a conventional combustion engine can only reach a range of 30% to 40% [48]. The wasted energy can be the source of power for maintenance-free wireless sensor nodes. The available energy for harvesting is thermal and kinetic energy. Figure 3a presents the energy audit of a traditional diesel engine using a pie chart [49]. It is understood that energy loss is inevitable during the operation of an engine resulting with the fuel efficiency being less than 50%. Most of the wasted energy is in the forms of heat and kinetic energy. For typical diesel engines in tested in 2009, the heat lost with the exhaust is approximately a quarter of the fuel energy. Meanwhile, exhaust emission is also a process in which gas flow may be used to generate force. Therefore, both thermal and kinetic energies can be harvested from the machine exhaust. Another form of the wasted energy is heat rejection (heat releasing into the environment with the coolant) with the energy supplied being slightly more than the exhaust. It is mainly a result of a significant increase in the temperature of the environment and components of the mechanical system, this provides an opportunity for powering WSN if there are effective and efficient thermal energy harvesting technologies. Although the development of technologies improved higher fuel efficiency for engines to around 50% in recent years (for example, Wärtsilä engine fuel efficiency increases year-over-year as shown in Figure 3b [50]), a mass of fuel energy still dissipates in the form of heat and kinetic energy. Nevertheless, since the transmission efficiency of machines is always less than 100%, most of the lost energy being used is due to overcoming friction and generated heat. Interestingly, researchers at the University of Huddersfield have analyzed the thermodynamics of an industrial helical gearbox of 10kW by thermography signals to obtain the heat distribution of the gearbox as shown in Figure 4b. It shows the thermal energy at the motor drive end and gearbox housing is relatively high and the temperature near the cooling system (in this case a fan) is much lower than shown in Figure 4a, this can be utilized to assemble a thermoelectric generator to power the gearbox monitor [51]. In addition, the cooling system of a machine can also generate useful kinetic energy while cooling the mechanical system. These examples illustrate that these different forms of wasted energy can be effectively harvested to power the wireless condition monitoring systems with little effect on the overall mechanical efficiency.

In summary, internal and external energy resources can be employed separately or simultaneously using various energy harvesting technologies or hybrid energy harvesters, such as a hybrid triboelectric-piezoelectric generator [52]. Solar energy can be scavenged to supply power for WSNs applied in the outdoor machine condition monitoring because of its abundance. Moreover, because of a considerable quantity of waste thermal and kinetic energy in mechanical systems, most machines have sufficient power resources for harvesting to achieve a WSN-based maintenance-free condition monitoring system.

## 3. Energy Harvesting Techniques and Applications

As discussed in Section 2, a more generalised real-time machine condition monitoring WSN system needs to deal with large amounts of data. The transmission of lower varying data, such as temperature, pressure and strain, gives valuable information for condition monitoring, which can be at low power levels. However, the main stream condition monitoring technologies still rely on the fast variation data such as vibration, acoustics and acoustic emissions, which need either high bandwidth transmission or high capacity local processing, which is generally achieved with higher power consumption. This means that power levels from milliwatt to watt range need to be made available for different scenarios. To meet these requirements, the operating principles of various EH techniques and typical efficient models or prototypes of generators, especially more applicable to mechanical systems, are revisited in this section. In addition, advantages and disadvantages of these technologies are summarized to provide detailed information for different applications in machine condition monitoring.

### 3.1. Light Energy Harvesting

**Figure 5:**
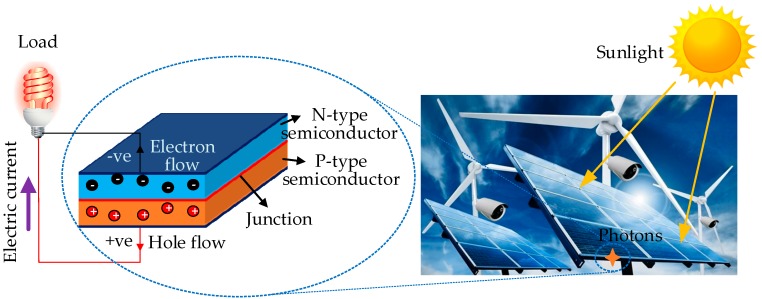
Working principle of photovoltaic cells.

Light energy, for example the energy from sunlight or artificial light, is generally renewable and ubiquitous energy source, which can be harvested and accumulated to power sensor nodes through the photovoltaic technique [53]. Photovoltaic cells manufactured with semiconductor materials can directly convert the light waves into electrical energy through the photovoltaic effect as illustrated in Figure 5. Free electrons and holes are produced at PN junctions under the influence of sunlight and then move to the N-type and P-type semiconductors to form a potential difference [54]. A current can be generated when an external conductor is connected to the electrodes of photovoltaic cells.

**Figure 6:**
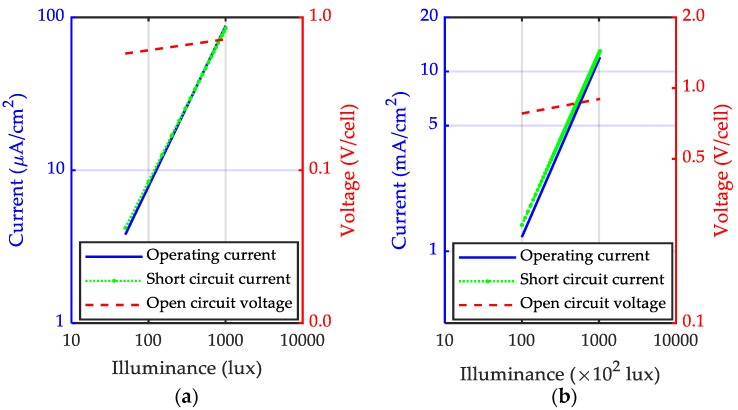
Relationship between the solar cell outputs and illuminance: (**a**) Indoor illumination; (**b**) Outdoor illumination.

The amount of energy harvested from light is related to the illumination level. For example, artificial light, usually an indoor light source, can only generate illumination at a low level of below 1000 lux. The magnitude of sunlight illumination level (up to 120,000 lux for brightest sunlight) is significantly higher than the artificial one. Figure 6 compares the relationship between the electric output and the illuminance level of Panasonic photovoltaic cells for indoor and outdoor illumination [55,56].

To improve the performance of photovoltaic cells, many researchers have contributed to the advancement of the photovoltaic materials. Photovoltaic cells are mainly classified into three types according to three types of materials: silicon (a commonly used semiconductor material), semiconductor compounds and novel materials [57]. Sampaio et al. [54], systematically examined the development of photovoltaic solar cells with different materials, the worldwide markets, as well as their advantages and disadvantages. Although conventional silicon solar cells dominate the commercial markets, a new types solar cells manufactured semiconductor compounds or novel materials, such as the thin film cells and organic photovoltaic cells, are being developed rapidly due to their characteristics of low cost, high conversion efficiency and flexibility, and low environmental pollution.

Because of the inconstant availability of solar energy, the direct use of electricity generated by photovoltaic cells or panels leads to low efficiency and inconvenience. Rechargeable batteries can be used to store solar energy for many applications. They are widely used in portable devices (such as watches and calculators) and outdoor applications (like solar street lights and solar heaters). Li et al. [58] have conducted a comprehensive survey on the advancement of solar-powered rechargeable batteries and characteristics of two distinctive connection methods: external combination with photovoltaics and internal integration with photo-electrodes. With the assistance of integrated technology and nanomaterials, small solar energy harvesting systems can be built to provide enough power for tiny sensor systems. Promising nanomaterials and nanotechnology applied for the solar energy harvesting have been discussed in [59]. Escobedo et al. [60] have designed the first printed flexible tag with characteristics of small size, low cost and low power consumption for gas concentration measurement in sealed environments, which can be powered by two miniaturized solar cells with the energy solely harvested from sunlight or bright artificial light.

Sunlight is virtually inexhaustible, renewable, clean and can be harvested to power large-scale outdoor machines efficiently and effectively. For example, it is prevailing for solar-powered aircraft systems in the aerospace industry [61,62,63,64]. Some scholars have reviewed its utilization powering water pumping systems instead of diesel power in agriculture [65]. Nevertheless, in solar energy harvesting, there are some major disadvantages such as instability, time-variance and regional restriction. inevitably affect the conversion efficiency, although some researchers have studied solar cells suitable for all weather applications [66]. For machine condition monitoring, micro-scale photovoltaic cells working with storage batteries can power the sensor nodes to monitor the condition variables of outdoor machinery such as vehicles, marine systems, mining machines and offshore equipment. Compared to sunlight, the conversion efficiency of artificial light is much lower due to the low intensity of the excitation source. Indoor light cannot yet supply enough power for WSNs on indoor machine condition monitoring, and only limited references mention the indoor light harvesting application [60].

Until major advances are made in photovoltaic technology it is only useful as a backup for other energy harvesting technologies for indoor machine condition monitoring. For outdoor use, it is undeniable that light energy harvesting is a feasible way to power machine condition monitoring systems with low noise, although solar energy harvesting is restricted by the region, weather, solar panel area, cost and maintainability. A solution is to include rechargeable batteries [67] to store the electricity generated by photovoltaic solar cells, which converts the electrical energy into the chemical energy for further storage and recyclable conversion [58]. Consequently, photovoltaic cells combined with batteries have the potential to implement online machine condition monitoring. More importantly, solar energy harvesting based condition monitoring system can increase reliability and safety and decrease maintenance costs. But the cost of materials and installation for photovoltaic cells and batteries are significantly expensive [68], which is unsuitable for condition monitoring of normal machines.

### 3.2. Electromagnetic Energy Harvesting

Electromagnetic energy is usually captured from the ambient RF sources which generate high electromagnetic fields, like TV broadcast stations, radar stations, Wi-Fi routers, Bluetooth, global system for mobile communications (GSM) and other communication networks [69,70,71]. RF energy harvesting devices can convert electromagnetic energy into a useful direct current (DC) voltage to power low-power consumer electronics and WSNs.

**Figure 7:**
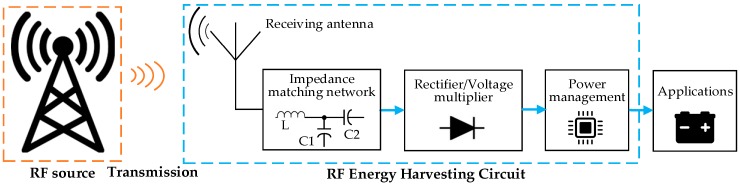
Structure of RF energy harvesters.

The radio signals have a wide frequency range from 300 GHz to as low as 3 kHz, which are used as a medium to carry energy in a form of electromagnetic radiation. A basic RF energy harvesting system consists of a receiving antenna, matching circuit, rectifier, and power management as shown in Figure 7 [72]. The antenna aims to capture electromagnetic waves, and then the matching circuit is used to achieve maximum power by using coils and capacitors. The rectifier circuit can convert the alternating current (AC) signal to a DC one, and finally the voltage output is adjusted using the voltage elevator [73].

**Figure 8:**
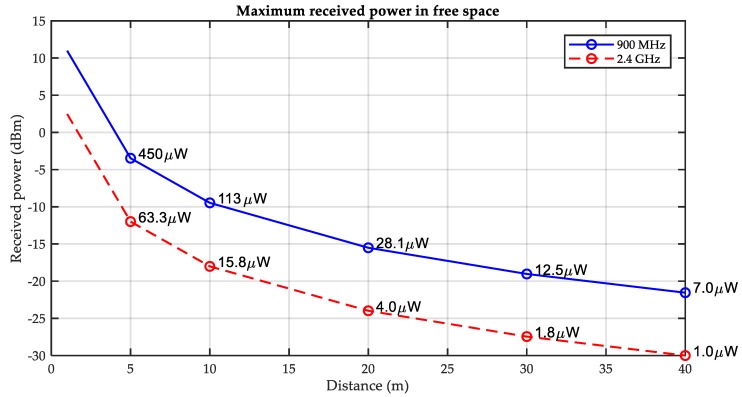
Relationship between received power and distance for RF energy harvesting.

Due to the wide coverage of RF, RF energy harvesting is suitable for powering a larger number of devices distributed in a wide area however the energy carried in the far-field RF waveform is limited [69,74]. According to the reference [69], the harvested power from different RF sources is between 1 μW to 189 μW at a frequency of around 900 MHz and a distance of 5 m to 4.1 km. The energy harvesting rate varies significantly depending on the source power and distance. The relationship between received power and distance in the environment of 900 MHz and 2.4 GHz is described in [73] as shown in Figure 8. Zeng et al. [75] summarizes and concludes the radiative wireless power transfer techniques and the novel methods to tackle the challenges (such as more compact antenna equipment, increasing conversion efficiency, minimizing impact on WSNs) in the period of the development of wireless energy harvesting. Popovic et al. [76] has successfully designed and manufactured a RF energy harvester integrated with all functions to harvested the 1.96 GHz and 2.4 GHz radiation energy. As with the development of WSNs, WSN-based Internet of Things (IoT) has improved dramatically, resulting in extended lifespans and a wider range of applications utilizing RF energy harvesting. Nguyen et al. [77] has designed a self-sustainable RF EH algorithm for IoT applications through adapting the EH period according to stochastic characteristics of the traffic load from IoT applications and the incoming RF energy at sensor nodes. Notably, a new relay policy in RF EH for IoT networks has been proposed by Behdad et al. to maximize the network effective lifetime in [78]. Moreover, RF energy harvesting has attracted the investigations on radio-frequency identification (RFID) which consumes low power to identify and track objects’ position with passive tags scavenging energy from nearby radio waves [79,80].

However, such RF signals are rarely used in the mechanical systems. The RF sources around machines are usually undesired and thus the potential energy harvested from these RF signals is limited. It is insufficient as a primary power source, but RF energy harvesters can assist other energy generators. Additionally, the RF energy harvesting technology still has a bright prospect due to its rapid development and wide application in wireless networks.

### 3.3. Thermal Energy Harvesting

In general, heat energy coming from temperature variation in the environment can be scavenged into electricity via some key thermal conversion techniques based on the Seebeck effect [81,82,83,84]. Thermoelectric energy harvesting and pyroelectric energy harvesting, relying on the temperature changing over distance and time, respectively, are the two most commonly used thermal energy harvesting techniques.

#### 3.3.1. Thermoelectric Energy Harvesting

**Figure 9:**
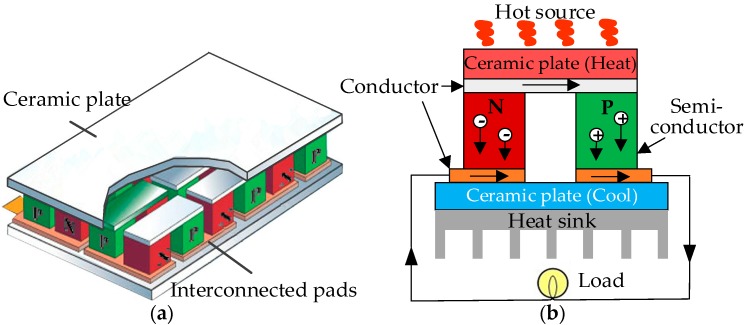
Thermoelectric generators: (**a**) Typical structure; (**b**) Seebeck effect.

Thermoelectric energy harvesting [85] is a conventional technique for converting wasted thermal energy into the electrical power by means of thermoelectric generators exploiting the Seebeck effect. A thermoelectric generator consists of a series of PN junctions connected end to end as shown in Figure 9a. Its aim is to increase the action area to increase the amount of the electricity harvested. Figure 9b gives the working principle of each PN junction. When a temperature gradient is established between the hot and cold ends of the ceramic plates, free electrons and holes will move towards their specific directions and generate a potential difference in proportion to the temperature difference between the N-type and P-type semiconductors. This phenomenon is known as the Seebeck effect.

Thermoelectric generators are widely utilized in wearable devices because the human body is a constant heat source with a temperature difference with the ambient temperature most of the time [86]. Wearable thermoelectric generators also contribute to clinical medicine through harvesting energy from the human body to support wearable electronics for health condition monitoring to avoid frequent battery replacement [87,88,89]. Since thermoelectric generator is a heat engine, its conversion efficiency is limited by the Carnot cycle efficiency, which is one minus the ratio of the cold and hot temperatures. The generated power is usually insufficient for the requirements of devices. To increase the power output, new thermoelectric generators modules are designed with compact structures [84,90]. Another approach is exploring new high-performance materials. For instance, some designers [91] have fabricated a flexible thermoelectric generator with special N-type and P-type thermoelectric materials relying on nanotechnology and obtained about 23.9 mW power at a 22.5 ℃ temperature difference. Because of its flexibility, this thermoelectric generator attaches to the heat source surface with an increase in the output power. This shows that the nanostructures of thermoelectric converters not only reduce volume and save materials but also effectively improve the performance of traditional thermoelectric generators. According to the advancement of the nanotechnology and fabrication techniques, numerous new promising nanomaterials and the optimisation of structures and circuits [92,93,94,95] have been investigated to improve the thermoelectric harvesters. Proto et al. [96] have provided a comprehensive review on nanogenerators for human body energy harvesting.

The machine in service is another heat source with temperature differences from the surroundings. Because of the features of safety, reliability and durability, as well as the extensive existence of temperature difference, thermoelectric generators have been widely applied in vehicles, aerospace, ships and other industrial operations [48,97,98]. Some of the generators aim to reduce fuel consumption and CO_2_ emissions by converting exhaust heat into an alternative energy as an electric power backup. Others are designed to provide electricity for WSNs using for structural health monitoring (SHM) or system condition monitoring of machines [28,99].

It is confirmed that spatial temperature gradients are available, continuous and stable in mechanical systems. More importantly, thermoelectric harvesters have characteristics of high stability, durability and safety. However, relatively low conversion is one of the disadvantages of thermoelectric energy harvesting. It is determined by temperatures of the cold and hot ends, as well as the figure of merit (ZT, properties of thermoelectric material). The maximum of ZT is about 2.6 leading to the maximum thermoelectric conversion rate of about 20% presently [100]. But the development of innovative structures, promising nanotechnology and emerging nanomaterials can promote the conversion rate to achieve high efficiency, flexibility and conformability in the field of machine condition monitoring. Another disadvantage in applying thermoelectric generators is that the temperature difference found in many machine systems is small, leading to small power gains. Even the usage of the heat sink can increase the temperature fluctuation, but the harvesting device may be too bulky and too difficult to install, which is a challenge in designing the structure of thermoelectric generators used for maintenance-free machine condition monitoring in the future.

#### 3.3.2. Pyroelectric Energy Harvesting

Compared with thermoelectric energy harvesting, an indispensable prerequisite of pyroelectric energy harvesting is temperature fluctuation over time [10]. The phenomenon utilised is that the polarization intensity of some dielectric materials releases charges with temperature fluctuations; this is called the pyroelectric effect [101]. Normally, free electrons and holes generated by the spontaneous polarization of a crystal are respectively neutralized by the holes and free electrons on the surface of the crystal. In certain materials, for example gallium nitride, the polarisation changes as the temperature changes and a voltage appears across the crystal. The conversion efficiency of pyroelectric energy harvesting is much higher than that of thermoelectric energy harvesting, especially using new temperature cycling techniques (like the Olsen cycle [102]). Hence, finding a source of time-varying temperature becomes one of the challenges of pyroelectric energy harvesting.

From the references examined, it is clear that researchers are striving to capture temporal temperature gradients in different fields. It is shown in El Fatnani et al. [103] that energy in the form of temperature fluctuations generated by infrared radiation coming from an infrared lamp can be harvested by the pyroelectric effect. This approach also contributes to the enhancement of the autonomous infrared sensors by means of pyroelectric materials. In addition, Ayesha et al. [104] has investigated a commercial patch transducer that can be a self-sustaining pyroelectric generator by taking advantages of temperature gradients caused by the process of breathing. This may be the potential applications on the non-invasive human healthcare monitoring. Similarly, pyroelectric energy harvesting is also appropriate for water splitting through harvesting temperature fluctuations and generating sufficient power [105,106]. In the field of chemistry, Zhao et al. [107] harvests energy from waste heat with a flexible pyroelectric device and successfully operates a temperature sensor to monitor this process in real time.

**Figure 10:**
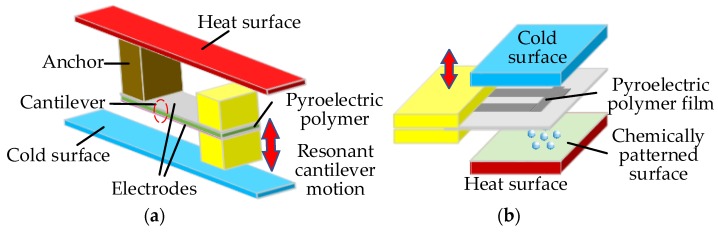
Schematics of typical pyroelectric generators: (**a**) Cantilever structure; (**b**) With liquid-based surfaces

In order to produce a greater temperature difference, some devices with different architectures are designed to directly capture the time-dependent temperature gradient from spatial temperature fluctuations through the movement of the pyroelectric materials. For instance, Figure 10a describes the schematic of a typical pyroelectric energy generator with a bi-metallic cantilever beam construction consisting of two metal electrodes of a capacitor and the pyroelectric dielectric material [108]. One end of the cantilever structure is connected to the hot surface via an anchor, and the other end is connected to two small masses. When the cantilever is heated and bends towards the cold surface, the structure rapidly loses heat and bends back towards the upper hot side. Then the structure rapidly heats and bends away from the hot side. This process subtly converts the spatial temperature gradients into temporal temperature fluctuations of the cantilever structure. To harvest more energy, millions of the millimetre sized converters can be fabricated in 2D arrays to create a much larger device. Figure 10b gives another pyroelectric harvester schematic designed by Cha et al. [109] with liquid-based switchable thermal interfaces for rapid cycling of the pyroelectric polymer film between hot and cold surfaces. Its power density is about 110 mW/cm^3^ at an operating frequency of 1Hz. Zhang et al. [110] adopts a similar structure to fabricate a thin-film pyroelectric generator with the maximal output power of 2.2 μW when the external load was 0.1 mΩ. After reviewing some papers relating to such structures, it is concluded that the challenge of pyroelectric energy harvesting is mainly focused on high-frequency temperature fluctuations and high efficiency of energy extraction cycles.

With the development of nanotechnology, thin films, nanowires and nanofibers have been investigated for pyroelectric EH, which gives the opportunity for pyroelectric techniques to be incorporated with other EH approaches [111]. Yang et al. [112] has fabricated the first pyroelectric nanogenerator with the pyroelectric ZnO nanowire arrays to convert the thermoelectric energy into electricity. The designed pyroelectric nanogenerator can drive a liquid crystal display directly and a light-emitting diode with the assistance of a lithium battery, which demonstrated that pyroelectric nanogenerators are the potential applications in self-powered WSNs [113,114]. Moreover, high-temperature resistant nanomaterials are appropriate for extremely harsh environments.

Start-up transients or load changes of rotating machinery can supply the temporal temperature fluctuation for pyroelectric generators, but these changing operating states only last a comparatively short time, and storage devices cannot rely on short-term energy collection for a long period of condition monitoring. Hence, thermoelectric and pyroelectric EH techniques mainly focus on the spatial temperature gradient (available in oscillating heat pipes [115], heat exchangers, marine boilers, engine, motors, pumps and compressors). For indoor machines, they play an important role in achieving self-powered WSNs for condition monitoring because of wasted energy existing in the form of heat and supplying sufficient energy for WSNs. Both thermoelectric generators and pyroelectric generators are maintenance-free and have the advantages of long lifetime, lightweight, durability and compactness. However, their cost is relatively high when compared to their power conversion efficiency. The development of nanomaterials may fill this flaw in the future.

### 3.4. Mechanical Energy Harvesting

Wasted mechanical energy widely exists in the environment, such as human motion, bridge vibrating, vehicle driving, ocean surging, wind blowing, and mechanical rotating. It is a green and sustainable energy source, which can be efficiently captured to generate useful electric power for self-powered devices. Various forms of kinetic energy induced by the motion of objects, elastic potential energy caused by the deformation of objects and electric potential energy resulting from conservative coulomb forces are the main mechanical energy sources found in industrial applications. The primary techniques of energy scavengers will be detailed, and some related research will be reviewed in this subsection.

#### 3.4.1. Piezoelectric Energy Harvesting

**Figure 11:**
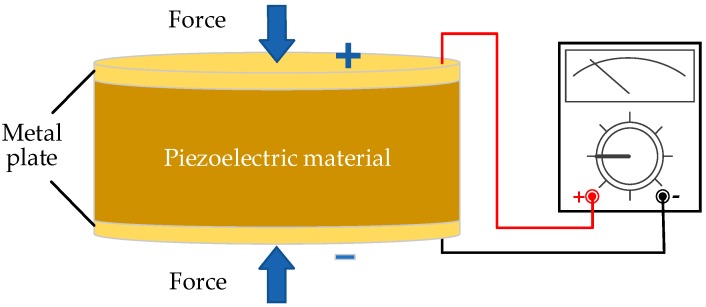
Direct piezoelectric effect principle.

Piezoelectricity, also known as the piezoelectric effect, is a phenomenon converting mechanical energy into electrical energy due to the inherent polarization characteristics of certain crystals [116]. Figure 11 shows its working principle that the positive and negative charges are produced on two opposite surfaces of the dielectric when the dielectric is deformed by an external force.

**Figure 12:**
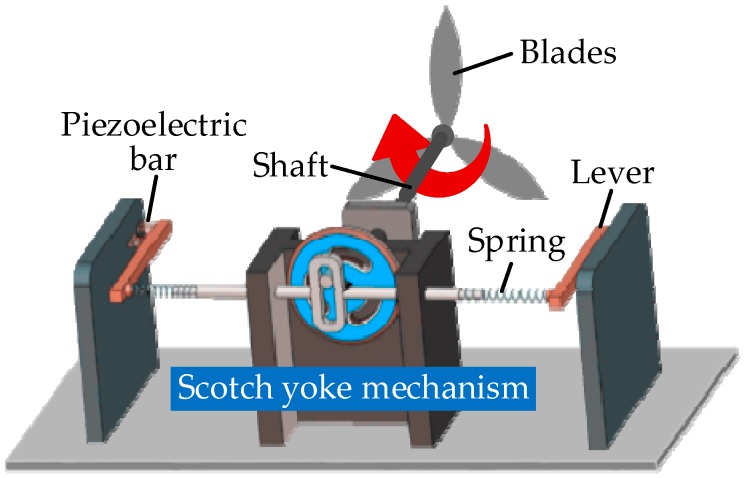
Schematic of a piezoelectric wind turbine: an internal structure of the device.

Piezoelectric ceramics and polymers are two common representative piezoelectric materials [117]. Piezoelectric generators can convert vibration energy to electrical energy. Piezoceramics have high conversion efficiency leading to the extensive applications in industry and research fields, but they are rigid with low ductility and toughness. Turkmen et al. [118] designed a cymbal type energy harvester with two different piezoelectric ceramics, PZT-5H and PZT-8, and showed that PZT-5H is most suitable for this design. Additionally, some designers have developed a piezoelectric-based electrical model and a prototype device with a cantilever beam structure to power a self-sustaining wireless sensor in heating, ventilation and air conditioning systems [119]. In [25], the kinetic energy of wind, extracted by a wind turbine, is successively converted into the rotational motion of a shaft and linear vibrations with a Scotch yoke mechanism [25]. Then, the vibrations are converted into electricity by the piezoelectric (PZT4)-lever design. The schematic diagram of this piezoelectric wind turbine is described in Figure 12.

Conversely, piezopolymers have been successfully utilized in the field of sensors, especially for biomedical sensors, because they are soft, deformable and lightweight, but their conversion efficiency is relatively poor compared with piezoceramics. Three types of portable piezoelectric devices designed with flexible piezoelectric materials by Mutsuda et al. [120,121] can harvest energy from ambient kinetic energy, such as water, wind and other mechanical vibrations.

In order to create energy harvesters with high flexibility and efficiency, piezoelectric composites made of piezoceramics and piezopolymers have had rapid development from the 1970s [122,123]. Presently, generators made by piezoelectric composites have a wide range of applications in medical, sensing, measurement and other fields. For example, some researchers have improved a piezoelectric generator with the linear cantilever beam (made by an innovative type of multilayer composite) instead of the lengthening beam to power WSNs in [124]. The power response of the system is up to 2.66 mW/g at a frequency of about 90 Hz, which has significant advantages (about 2.5 times higher) over the classical piezoelectric energy harvester. Wang et al. [125] designed a generator with composite materials to harvest friction induced vibration energy, which offers a new approach to scavenge the wasted vibration energy.

To further increase the overall harvesting efficiency of piezoelectric generators, Todaro et al. [126] makes a survey on the current status of micro piezoelectric energy harvesters and focused on two primary challenges, the frequency bandwidth and the conversion efficiency. These challenges also attract the attention of other engineers. For example, a piezoelectric energy harvester is designed and fabricated with two beam structures to bring the second mode frequency closer to the first one with the assistance of a variable cross-section and a tapered cavity to broaden the resonance bands [127]. Orrego et al. [128] has fixed a flexible piezoelectric membrane with self-oscillations in an innovative inverted flag to scavenge wind energy for supporting self-powered devices. For achieving high efficiency of energy harvesting, nanomaterials have been investigated. Briscoe et al. [129] have made a survey of the development process of nanomaterials and nanostructures. Developers from the Chinese Academy of Sciences have designed a nanogenerator with an extra wide response bandwidth of a single cantilever by adding a limiter at one side of the cantilever, and further widened the bandwidth through combining a series of cantilevers with different response bandwidths [130]. Additionally, an innovative MEMS-based energy harvester is explored and fabricated by the integration of strain sensitive nanocomposite materials and MEMS devices in [131].

A potentially fatal flaw of piezoelectric materials is that the performance of materials will gradually degrade, leading to lower conversion efficiency and lower power output with the time. Wireless condition monitoring systems powered by the converted electricity are prone to failure because of the insufficient power supplied. 

The few suitable materials and the complicated fabrication process are the key problems that need to be solved before piezoelectric generators become an efficient commercial product. Furthermore, the improvement of piezoelectric generator structures is a tremendous challenge aiming at selecting its resonant frequency in the frequency band with energy concentration or working at a wide resonance bandwidth. In a word, the piezoelectric EH technique has a bright and promising future in machine condition monitoring if the aforementioned challenges can be addressed.

#### 3.4.2. Electromagnetic Energy Harvesting

Electromagnetic induction refers to a part of the conductor in a closed-circuit which cuts magnetic flux in a non-parallel direction to generate an induced current in the circuit. In other words, the wasted kinetic energy can be converted into electricity based on the conversion mechanisms with the assistance of magnetic fields for small-scale autonomous powered devices.

The disadvantages of electromagnetic generators are the mismatching of electromagnetic generators’ resonance frequencies with external vibration sources and the narrow response frequency band. As the external vibration source is random and non-stationary, the output power will be dramatically decreased if the matching frequency has a slight offset [11]. A mathematical model is built by Bernal and García [132] to study the interaction between electromagnetic induction and damping, and also the relationship between dimensions and energy output. Meanwhile, Cooley has analysed and verified the mechanisms and dynamics of vibrational electromagnetic energy harvesters by means of matrix operator form representing the eigenvalue problem of harvesters [133]. The results show that eigenvectors with strong electromechanical coupling usually generate large average power output. During the circuit design process, it should be paid attention that the inductance, resistance, and capacitance have effects on the eigenvalues and dynamic response of harvesters. For further understanding of the electromagnetic energy harvesting, Naifar et al. [134] summarizes a substantial amount of literature published during recent years and gives details of the principle behind electromagnetic generators. They present many electromagnetic prototypes and classify them by the different harvester architectures (like the cantilever, magnetic spring and rotary pendulum). 

Table 5 lists several electromagnetic generators designed in these years and compares their performance based on power density and features. It is reported that most of the generators work in a narrow and low-frequency band. Moreover, the magnitude of the power density varies greatly, even for those that approximate the response frequencies and accelerations of the energy source.

From the references, most of electromagnetic generators can convert kinetic energy into a considerable amount of energy (generally at milliwatt level) at a low-frequency band of vibration with relatively low fabrication cost. However, the increase of the internal resistance caused by the micro-coil leads to inevitable power consumption, which may affect the output power of the electromagnetic generators. In addition, the size of the coils makes the microscale electromagnetic generator fabrication difficult to implement at present. There are also obstacles to integrate them with MEMS, which restricts the widespread applications of electromagnetic generators.

#### 3.4.3. Triboelectric Energy Harvesting

**Figure 13:**
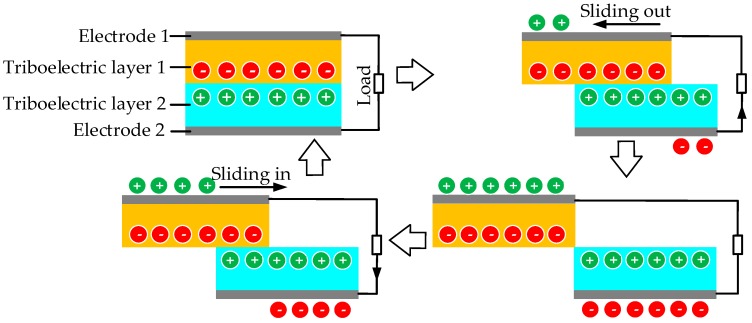
Working principle of the triboelectric effect.

Two objects manufactured with different materials can generate electron flow in a specific direction when they periodically contact or separate from each other by external forces. This electricity production process is called the triboelectric effect which is illustrated in Figure 13 [144]. External mechanical energy can be converted into electricity based on the triboelectric effect. Wang, in his reviewer paper [145], gave a particular table of triboelectric series which shows the order of various materials arranged according to their ability to donate or accept electrons in his review.

The triboelectric EH technique was developed later than other technologies. Many engineers have investigated triboelectric generators because of the ubiquitous ambient motions (such as wind or water flow [146,147,148] and human motion [149,150]) and generators’ characteristics of scalability, reliability, high efficiency and low cost [151,152]. Ref [153,154] describe two triboelectric harvesters designed and fabricated with similar structures, an integrated Zigzag shape and a stacked X-shape,. The power density can reach up to 27.96 mW/cm^2^ and 542.22 μW/cm^2^, respectively. Additionally, an ultra-soft and cuttable paper-based harvester is made with cheap commercial materials to scavenge the energy associated with the body’s motion, sound energy and wind energy in [155]. Another powerful triboelectric generator with the compressible hexagonal structure is designed and installed inside the car tire to power a wireless tire pressure sensor by Guo et al. [156].

**Figure 14:**
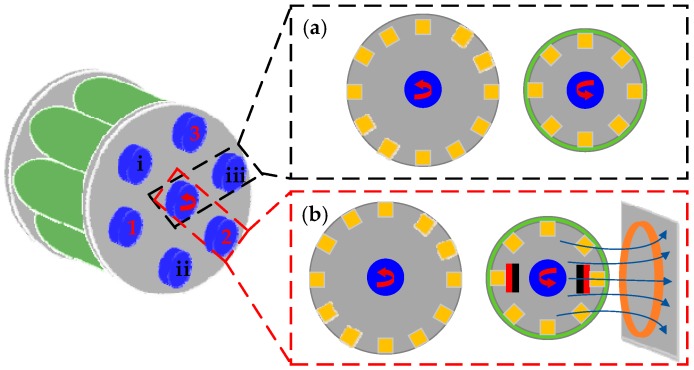
Structure of the designed hybridized generator: (**a**) Triboelectric generator unit; (**b**) Hybrid unit.

Moreover, some researchers have found success in triboelectric harvesters using rotational energy because of less abrasion of materials. For example, Xie et al. [157], Zhu et al. [158] and Ahmed et al. [159] designed three different rotating triboelectric generators respectively and all three prototypes show high performance in harvesting wind energy. A novel linear-grating triboelectric generator is proposed by Zhu et al. [160], which can be manufactured as three different configurations to harvest energy from rotation, rolling and linear piston motion. Yang et al. [161] describes a model designed for rotating machinery. Figure 14 shows its architecture consisting of six contact-separation mode triboelectric units shown in Figure 14a, and three electromagnetic and triboelectric hybrid units shown in Figure 14b. The fabricated prototype works with the gear transmission structure to implement EH on the rotating machines. Generally, triboelectric energy harvesting is combined with other technologies to capture enough energy to support the actions of wireless monitoring systems, such as [162].

From the references, it is apparent that triboelectric harvesters are normally designed as nanogenerators with advantages of robustness, easy integration, high conversion efficiency and low cost. However, the friction and the inevitable wear of the two triboelectric layers results in a limited lifetime, also rotating contact can reduce the material losses to some degree. The wear of materials leads to lower conversion efficiency and gradually cannot meet the energy requirement. Furthermore, heat generated by the wear may cause catastrophic accidents in some environments.

#### 3.4.4. Electrostatic Energy Harvesting

**Figure 15:**
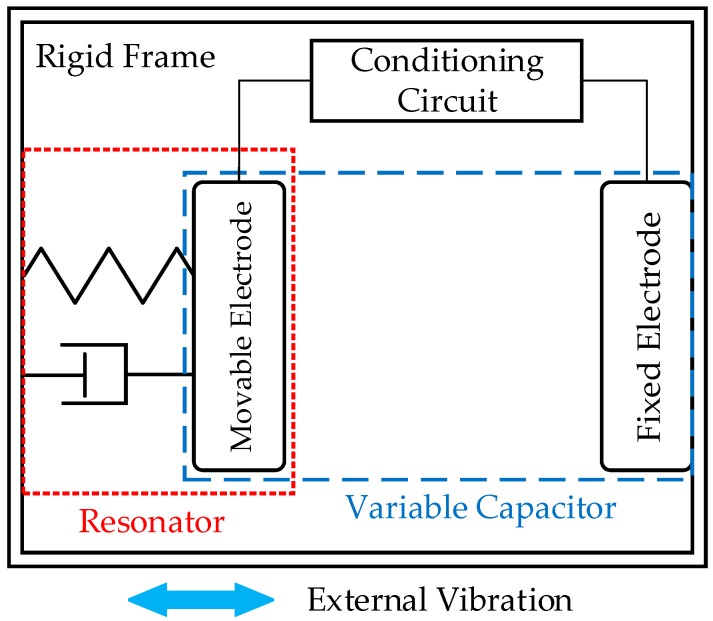
Schematic of an electrostatic energy harvester.

The working principle of the electrostatic EH is based on the variation of the capacitance, which converts mechanical energy into electrical form. The electrostatic EH is mainly achieved by using a parallel plate capacitor with one plate fixed and the other one movable. Consequently, the electrostatic EH can be more specifically named electrostatic kinetic EH, which mainly harvests the vibration energy in the machines. The vibration can provide great opportunities for EH since these are present in all machines to some degree. Generally, the electrostatic EH device consists of a resonator, a variable capacitor and a conditioning circuit, which is shown in Figure 15 [163].

To activate the electrostatic generator, the charge is injected into the capacitor at maximum capacitance and pulled off at minimum capacitance. The change of the gap between the electrode of the variable capacitor can generate the electric outputs. The mass of the movable electrode, the spring and the damper form a resonator. The resonator can constrain the motion of the movable electrode of the capacitor and capture the external vibration at a certain range of the frequency band in high efficiency, usually at the natural frequencies of the resonator. The implementation of electrostatic generator usually needs a DC to DC converter for extracting energy and furthermore, the operation of the converter has to be carefully synchronized at certain instants of the vibration cycle.

The electrostatic generators can be widely applied in various scale machines. Numerous applications of the electrostatic EH have been developed to harvest the mechanical vibration energy. Most of the electrostatic generators are based on a single variable capacitor. The electrostatic EH working principle shows that the harvesting system requires an external power source to polarise the capacitance or an electret material that induces charges spontaneously [163]. A passive electrostatic device can be applied as an auxiliary to an external power supply, but it is difficult to implement a maintenance-free sensor node [164]. With the electrets, the drawback is that charges cannot exceed a maximum threshold as it will reduce the conversion efficiency and retain the charges for a limited time. With advanced MEMS technology, some designers [165,166] have investigated and manufactured electret-based electrostatic generators, but only achieving microwatt power levels. Miyazaki et al. [167] has proposed an electrostatic generator which delivers 120 nW energy at the conversion efficiency of 21% with the excitation frequency of 45 Hz. Yen and Lang [168] have developed an asynchronous diode-based charge pump-based electrostatic harvester, which can generate 1.8 μW outputs. To overcome the pre-charging problem, the idea of the ‘‘doubler of electricity’’ or ‘‘Bennet’s doubler’’ is adapted to eliminating the need of batteries or other devices for pre-charging [169,170,171]. Although the researchers have designed various circuit structures to exclude the external power supply requirements, the low output power means that electrostatic EH technology cannot be used alone in the process of self-powered WSN-based machine condition monitoring.

Through making a survey on various techniques and prototypes for mechanical EH, it is shown clearly that structures of mechanical energy harvesters are complicated and with finite lifespan caused by the deformation and abrasion. In addition, the vibration energy is limited and most of the rotational energy is not wasted energy. Therefore, the mechanical EH harvesters are suitable only as auxiliary for thermal EH devices to supply power for WSNs.

### 3.5. Hybrid Energy Harvesting

EH technologies are classified based on different energy sources. Their working principles and generator prototypes investigated by researchers have been detailed above. The pros and cons of the aforementioned generators are thoroughly summarized and compared in Table 6 and Table 7.

To obtain sufficient power to implement self-powered or maintenance-free devices, multiple types of energy harvested by hybrid technologies have become the recent trend with the advancement of nanotechnology and fabrication technologies. The combination of different technologies can enhance their merits and overcome shortfalls.

A common improvement is the combination of two technologies. For instance, Bito et al. [172] packages a hybrid and efficient generator to convert room light irradiation and electromagnetic waves into electricity to power internet of things (IoT) wireless sensors with the assistance of a solar cell and RF energy harvesting techniques. Additionally, Guo et al. [173] designed a waterproof generator based on triboelectric and electromagnetic mechanisms which can be applied in harsh environments. A hybrid triboelectric and electromagnetic generator fabricated by Zhu et al. [174] can generate microlevel power density when it operates within a broad bandwidth (10-45 Hz). Similarly, for harvesting automobile rotational energy from the brake, Han et al. [175] has explored a generator with a six blade structure which uses triboelectric and electrostatic induction under the disc contact during the braking action and non-contact modes, respectively. A triboelectric generator can also be combined with a piezoelectric one. Yang et al. [52] has simulated a hybrid triboelectric-piezoelectric harvester model and analyzed the effect of parameter setting on maximum power output. This study can support the guidance of effective and efficient harvester design in future. Moreover, combining piezoelectric and electromagnetic mechanisms, the authors in [176] have invented and fabricated a hybrid generator by calculating the model parameters in advance. The generator can scavenge adequate power (up to 2.214 mW) from bridge vibrations and the surrounding wind flow to support the operation of WSN for bridge health monitoring.

In the literature, there are numerous examples of the integration of more than two EH technologies. For instance, the magnetic, thermal and mechanical energy were scavenged by electromagnetic, thermoelectric, and piezoelectric techniques implemented in a hybrid scheme to supply enough power for power grid condition monitoring using ZigBee sensor nodes [178]. Triboelectrification, piezoelectricity and electromagnetics were incorporated to achieve a hybrid nanogenerator with high sensitivity, efficiency and stable power consumption for transmission of temperature and vibration signals [27].

Hybrid energy harvesters provide reliable and stable power backup support for the self-sustainable WSNs. But the implementation of a hybrid energy harvester, like other individual energy harvesters, requires a power management circuit and should have a multiple-input power converter [179] to obtain a regulated desired voltage level to power WSNs and store excess electricity. Because different sources with EH technologies produce voltages and currents with different characteristics, such as a thermoelectric generator (which produces very low output voltages [180]) or a piezoelectric generator (which provides sinusoidal sources and needs to be rectified [181]). Some researchers have explored various effective integrated circuits of AC/DC or DC/DC converters. For example, a converter is developed for working on the condition and combination of energy generated with photovoltaic and thermoelectric harvesters [182]. Another converter is designed to regulate and combine the energy harvested with piezoelectric EH and thermoelectric EH technologies to successfully support the operation of ECG processor in wearable electronics in [183]. Dini et al. [180] has investigated an autonomous power converter which can be used for single or multiple sources and achieved the conversion efficiency up to 89.6%. In general, most converters implement single-source voltage conversion and energy storage. Converters used for multi-source energy harvesting should be designed according to the requirements because the conversion efficiency of autonomous converters is not high. As a result, the improvement of power management converters is beneficial for the application of EH for self-sustainable WSNs in machine condition monitoring.

As mentioned previously, with the drive of the hybrid EH technique integration and the limitation of the device volume, nanogenerators are a promising industry developing at a rapid pace. They are expected to provide effective and efficient solutions for self-powered WSNs due to the characteristics of flexibility, lightweight and integrability [59,184]. The statistics made by Wang et al. [185] reports that nanogenerator technology is rapidly developing as an emerging technology in China. Chen et al. [186] has devised and made a triboelectric-piezoelectric nanogenerator based on flexible fibres to attach to a soft surface to harvest kinetic energy. However, some critical challenges have a significant influence on large-scale industrial production and applications, such as the acquisition and lifespan of nanomaterials, development of nanotechnology, optimization of the nanostructure and conversion ratio of nanogenerators. There are lots of difficulties and barriers waiting for solutions during the development process of nanogenerators.

## 4. Wireless Sensor Network based Machine Condition Monitoring

Failure induced by various factors like improper installation, unsuitable temperature, corrosion, abrasion, fatigue, oil debris and occurred on components (such as motors, generators, engines, pumps, bearings, gears and shafts) of engineering systems can unexpectedly cause machinery to collapse and lead to significant losses for industries [1]. Therefore, the maintenance of machinery plays a vital role in industries and has been studied by researchers for decades. There are three common maintenance strategies arranged in order of progression: breakdown maintenance, preventive maintenance and predictive maintenance [187]. Breakdown maintenance is an unplanned maintenance way to maintain the damaged machines through the component repair or direct replacement, which seriously affects the continuity of industrial production and brings dramatically high maintenance costs. As a planned maintenance form, preventive maintenance is carried out according to the regular and periodic inspection and maintenance to detect and avoid issues before failures or breakdowns happen. Although this approach reduces the frequency of catastrophic failures and increases the reliability of systems, the operation of setting an optimal inspection period is complicated and frequent change of components is costly. In order to improve the stability of mechanical systems and reduce the economic losses and costs induced due to failures and breakdowns, an intelligent and effective maintenance strategy, predictive maintenance, needs to be established to prevent the occurrence of failures by relying on many techniques, such as thermal imaging, lubricant analysis and vibration characteristics [188].

Condition-based maintenance (CBM) is an effective and efficient strategy of predictive maintenance. It relies on the assessment of machine operating condition to determine whether the components require maintenance or not to enhance the stability and reliability of mechanical systems, minimise the labour and material resource costs, as well as improve the operating safety. First, raw data sets representing the condition of machinery (like temperatures, pressure, torques, vibrations, displacements, acoustics and acoustic emission) are collected by a variety of sensors (like thermocouples, pressure gauges, microphones and accelerometers) and saved in the storage devices through data acquisition devices. Then, the data sets are processed to present the machine conditions by various techniques. These signal processing methods in condition monitoring mainly consist of feature extraction, modelling and machine learning as shown in Table 8. Finally, decision making can give the recommendation of maintenance actions to prevent deterioration through fault diagnosis which is a process of determining health, faults and even their severity of systems being monitored.

CBM has advantages of enhancing machinery reliability, minimizing the maintenance time, saving the labour costs, improving the operating safety and guaranteeing the work continuity through reducing unscheduled downtime caused by catastrophic failures. But, the cost of purchase and installation for monitoring hardware (like acoustic emission transducers, piezoelectric sensors and data acquisition devices) and software is expensive. To solve this problem, WSNs have been focused and considered to utilize in the machine condition monitoring area because the sensors and wireless sensor nodes are economical, easy to operate, programmable, integrated, portable and with small sizes and low consumption. Additionally, the application of WSNs can avoid the cumbersome cable distribution and harsh environment restrictions and play an essential role in the realization of remote real-time condition monitoring of mechanical systems. 

However, WSNs have a fatal drawback compared with the conventional wired condition monitoring systems that the prevalent application of WSNs in machine condition monitoring is limited by the finite lifespan and capacity of traditional batteries. It is inconvenient to recharge or replace the exhausted batteries in a remote or inaccessible position, and it leads to the machine condition monitoring interruptions and wastes the labour and resources. Therefore, the wasted energy from machines or surrounding environments is attractive for researchers to harvest for powering the sensor nodes with EH technologies introduced previously. This approach substantially prolongs the life of sensing nodes and reduces maintenance costs of the monitoring system and the environmental contamination.

The potential recyclable energy sources in or around mechanical systems and corresponding EH technologies have been discussed in detail in Section 2 and Section 3, respectively. Several examples for various EH technologies are successfully applied in the WSN-based machine condition monitoring and these representative cases are demonstrated as follows to illustrate their prospects in self-powered WSNs in remote online machine condition monitoring.

As the external energy sources of mechanical systems, light energy and RF energy are seldom harvested in the period of machine condition monitoring. The photovoltaic EH technology is primarily applied for large-scale power generation and infrequently used for machine condition monitoring at present. Only few references can be reviewed here. Wang et al. [192] designed a multi-source (vibration, heat and light) energy harvester with piezoelectric, thermoelectric and photovoltaic EH technologies to power the WSN based acoustic emission sensing systems to successfully monitor the condition of the fear of a gas compressor. The manufactured prototype can generate 1.56 mW and 3.37 mW from 0.048 g vibration energy and thermoelectric energy of 62 ℃, respectively. Some researchers have investigated a WSN to detect the acceleration of the helicopter with the sensor nodes fed by the electricity harvested from the environment by a commercial RF energy harvester [193]. The distance between the tower antenna and the helicopters is about 76 m and the output DC power is about 70 µW. As a result, the solar energy is suitable to be harvested to power large-scale outdoor machines efficiently and effectively. The indoor light EH and RF EH can be considered as the supplement of other EH technologies.

**Figure 16:**
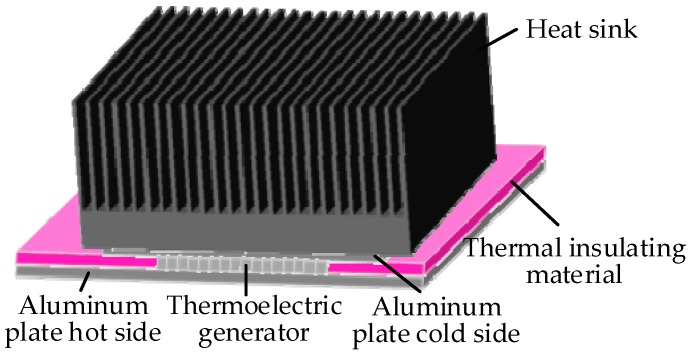
Schematic diagram of the designed thermoelectric generator device.

The internal energy sources are available and widely utilized for machine condition monitoring. For example, researchers at the University of Huddersfield designed a thermoelectric generator device, shown in Figure 16 and installed on the surface of the gearbox housing in Figure 4b, to harvest energy from the temperature gradient between the housing of the gearbox and the ambient temperature for powering a WSN system [51]. The temperatures of the ambient, gearbox surface and oil were simultaneously captured for the gearbox condition monitoring and fault diagnosis. Wu et al. [194] has established a mathematical model for evaluating the performance of the segmented thermoelectric generator by considering multiple parameters and then analyzed its efficiency and feasibility on a gas turbine operating at high temperature. After that, they designed a prototype with the segmented thermoelectric generators based on theoretical analysis to continuously and steadily power the sensing and monitoring system with the energy harvested from the hot surface of the gas turbine.

According to the aforementioned analysis of mechanical kinetic energy sources, several application examples of the piezoelectric generators are cited to validate the effectiveness and availability in machine condition monitoring and fault diagnosis. In 2005, Plessis et al. [195] has evaluated the output power of a packaged piezoelectric generators with the cantilever architecture and monitors machinery health condition with wireless sensor nodes powered by the piezoelectric generator. In the same period, Discenzo et al. [196] has proposed a self-powered WSN to monitor shipboard fluid pump condition in a terrible environment. A piezoelectric harvester is made to charge two lithium batteries to support the acquisition of vibration signals at 1 kHz and subsequent data processing and transmission. In addition, a piezoelectric generator is designed and tested both in the laboratory and on a large-scale turbo generator [197]. However, the result is not satisfactory because the power (microwatts) obtained on the real turbo generator are much lower than the power (milliwatts) collected in the laboratory. This conclusion explains that the resonant frequency of the piezoelectric generator system should be consistent with that of the mechanical system for optimum performance.

**Figure 17:**
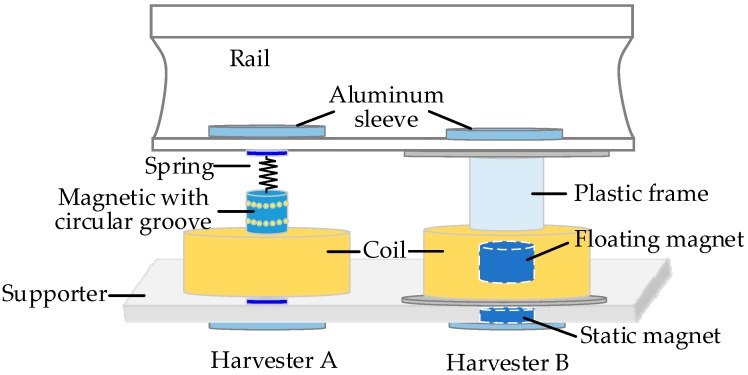
Electromagnetic energy harvesters: resonant Harvester A and magnetic levitation Harvester B.

Then, some electromagnetic generators applied in the field of machinery are focused to be introduced and discussed. In 2013, Waterbury and Wright [198] have investigated an electromagnetic generator, converting electricity from vibrations of large industrial pump motors, to support a self-sustaining wireless condition monitoring system. Another electromagnetic generator was made to scavenge rotating kinetic energy for powering a long-lasting CC2420 with a ZigBee module used for condition monitoring of the metal milling-processes and cutters in [199]. In addition, two types of electromagnetic generators are designed based on resonance and magnetic levitation respectively by Gao et al. [200]. The two electromagnetic generators are compared in Figure 17. They are installed on the railway and harvested energy industrial pump motors, to support a self-sustaining wireless condition monitoring system. Another electromagnetic generator is made to scavenge rotating kinetic energy for powering a long-lasting when the train passed through. Additionally, the designers have further studied how to achieve a maintenance-free wireless monitor system with the assistance of a magnetic levitation harvester [201]. Another successful case for monitoring the condition of induction machine rotor is designed by Fernandes et al. [202], and the fabricated magnetic generator successfully powered the NRF24L01 module for transferring the temperature characteristic of the motor.

**Figure 18:**
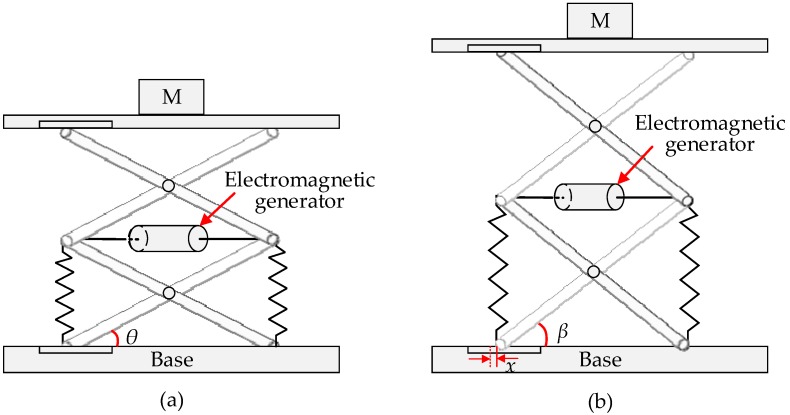
Schematic diagram of the designed nonlinear energy harvesting system: (**a**) State 1; (**b**) State 2.

Compared to the generators operating at extra low frequencies, another electromagnetic generator with the resonance at a fixed frequency of 200 Hz has been explored by Syed et al. [203] and installed on an industrial centrifugal pump to successfully power wireless sensors for condition monitoring. In order to overcome the narrow resonance frequency bands of electromagnetic generators, Wei et al. [204] designed a nonlinear vibration-based EH system model with a first order lever structure relying on an electromagnetic generator. The in-plane schematic representations of two consecutive operation states are shown in Figure 18. The advantage of this design is that it may be tuned to a particular source and it has the ability to collect random noise energy. The main disadvantage of these models is the large and bulky volume.

Some investigators are committed to collecting wasted mechanical energy with multiple technology integration. An integrated generator, consisting of six acrylic hexagonal prisms, a cylindrical mass and a magnet (working as six triboelectric harvesters and an electromagnetic one), is designed and installed on an automobile wheel to convert rotational energy of the wheel into electricity in [205]. In addition, some engineers designed a system to harvest vibrational, thermal and light energy for establishing a rotating machine monitoring system based on acoustic emission signals [184]. ZigBee was selected as the transmission technology in this instance.

Most of the models reviewed in this section are designed for monitoring the condition of machine systems with self-powered WSNs. However, the application of EH technologies in other fields is much more practical and prevalent. These designs can give some inspiration for applications of the individual or hybrid EH technologies in machine condition monitoring systems. It can be confirmed that ZigBee is utilized as a transmission method at a higher reliability now. But with the development of Wi-Fi and BLE, especially BLE 5, they will dominate in the market of machine condition monitoring in the future because they have advantages of low power consumption, fast transmission speed, long transmission distance, low cost and high stability.

## 5. Challenges and Future Research

Aimed at developing a generalised or configurable WSN based machine condition monitoring systems, this review has revisited different up-to-date energy harvesting principles and prototypes along with an examination of their advantages and disadvantages in terms of energy capacities, fabrication and efficiency. It was found that their performances including harvesting capacities depends on materials used, structural designs and energy resources available. In order to increase the power capacity harvested by effective generators using different technologies, the challenges are summarized in the following five points.

- The first challenge is the selection and optimization of the WSN. This is the trade-off between data processing and transmission in a WSN node. The advent of BLE 5.0 brings an opportunity for large data transmission with long transmission distance and low power consumption, as well as Wi-Fi. The design of WSNs is not limited to using ZigBee in the future. The ultra-low-power MEMS components with data acquisition, transmission, processing and other functions, as important components, should be improved further.
- The discovery and selection of energy sources that can be exploited with energy harvesters in mechanical systems is the second challenge. Although the recoverable energy in mechanical systems has been analysed in this paper, the energy losses, mechanical structures and environment for various types of machines are different. This obstacle increases the difficulty of the energy harvesting estimation. It is necessary to design adaptive energy harvesting devices or systems for a variety of applications of monitoring different machines.
- The next challenge is the improvement and optimization of EH technologies and devices. The energy loss during mechanical operation is mainly dissipated by thermal energy. Both the spatial and the temporal temperature fluctuations are small, and the conversion efficiency of thermal EH technologies is relatively low. Hence the thermal energy harvesting technologies need to be improved to reduce the limitation of temperature cycling techniques and to obtain more effective and efficient energy conversion. Because of the drawbacks of each EH technology, it is necessary to continuously improve the performance of the EH devices by the structural optimization of the systems. For example, it is challenging to design a harvester with an adjustable and flexible structure that can select the optimal frequency band according to the energy distribution of a mechanical system in the frequency domain.
- The design and optimization of power management circuits is also an important challenge because improving the conversion efficiency of the circuits can effectively and efficiently increase the DC output power, especially for the multi-source energy harvesters.
- The last challenge is the application of nanomaterials and nanotechnology on energy harvesting (nanogenerators). The emergence of nanomaterials has contributed to the integration of multiple energy harvesting technologies to increase the amount of electricity collected. With the help of nanomaterials, micro-scale wireless sensor nodes can be located inside the machine to acquire more accurate signals because the sensor is closer to the potential fault source and the effect of noise is reduced. However, nanomaterials also face the abrasion and performance degradation of materials like other conventional materials. Therefore, researchers should continue to make efforts to improve the durability and efficiency of nanomaterials to extend the lifespan of the harvesters and realize real maintenance-free WSNs for machine condition monitoring.

## 6. Conclusions

This paper gives an overview of a comprehensive range of promising energy harvesting technologies and systems for achieving self-powered WSNs in machine condition monitoring. After discussing the characteristics of the traditional wireless transmission technologies and power consumption of some typical modules, it is evident that ZigBee is prevalent in self-powered WSNs presently. But Wi-Fi and BLE are becoming more competitive as their rapid development. The internal and external energy sources for mechanical systems employed separately or simultaneously have the ability to provide enough electricity for self-powered WSNs in machine condition monitoring. Photovoltaic cells can be applied to outdoor machines and RF energy harvesters mainly work with other types of harvesters or for ultra-low power devices. The thermal energy harvesters, specially designed with nanomaterials and nanostructures, are considered to be the main EH devices to feed WSNs for achieving maintenance-free machine condition monitoring because a large amount of energy converted into heat energy existing in mechanical systems. Mechanical energy harvesters are commonly used in WSNs in various fields due to their features of high efficiency and low cost. However, their structures are complicated and have a finite lifespan caused by the deformation and abrasion. Therefore, the mechanical EH harvesters are suitable as the auxiliary for thermal EH devices to supply power for WSNs. The pros and cons of different energy harvesting technologies have been made clear. Lower conversion efficiency is a major limitation of thermal EH technologies. Additionally, the abrasion and performance degradation of materials becomes a crucial challenge to mechanical energy harvesting techniques. As a result, hybrid energy harvesters are widely investigated by researchers and will dominate the market in the future. In addition, the design and optimization of power management circuits should be considered to improve their conversion efficiency to effectively and efficiently increase the DC output power and supply sufficient power for WSNs.

In terms of the development of energy harvesting technologies in machine condition monitoring, the future research mainly focuses on the development of adaptive harvesters, as well as the durability and efficiency of nanomaterials or nanogenerators. Fabrication technology and nanotechnology offer an opportunity to integrate the energy harvester with ultra-low power WSNs to monitor accurately the condition of machines online.

## Figures and Tables

**Table 1:** Comparison of traditional wireless transmission technologies.

Technology	Transmission Rate (bps)	Transmission Distance	Power Consumption			Features
			Sleep (μW)	Transmit (mW)	Receive (mW)	
Low power Wi-Fi (802.11g) [31]	54 M	1 km	300	350	270	High speed, high power consumption and high reliability.
BLE 5.0	2 M	Up to 300 m in theory	-	-	-	High speed, long distance, wide bandwidth, ultra-low power consumption and high compatibility.
BLE 4.2 [32,33,34,35]	1 M	Up to 100 m, Normally operate within 10 m	8	60	53	Low power consumption, low cost, high security and low latency.
ZigBee [31,32,33,34,35]	250 k	10 to 100 m	4	72	84	Low power consumption, low cost, low complexity and self-organization.
EnOcean [36]	125 k	Up to 30 m	0.60	99.0	72.0	Energy harvesting based and ultra-low power consumption.
Z-wave [33,36]	40 k	Indoor: 30 m or 40 m Outdoor: 100 m	3	70	65	RF-based, low cost, low power consumption, low radiation, anti-interference and high reliability.
ANT [33,34]	60 k	30 m at 0 dBm	3	110	75	Utra-low power consumption, high flexibility and proprietary.

**Table 2:** Typical wireless communication modules and parameters.

Wireless Transmit	Module	Microcontroller Unit (MCU)	Data Rate (bps)	Power Consumption		
				Sleep (mW)	Transmit (mW)	Receive (mW)
Wi-Fi (802.11g)	ESP8266	32-bit Tensilica L106 @ 80 MHz or 160 MHz	72.2 M	45	420	168
	CC3200 Wi-Fi Subsystem	32-bit ARM Cortex-M4 @ 80 MHz	54 M	43.92	990	201.6.4
	SPWF01SA	32-bit STM32 ARM Cortex-M3 @ 120MHz	54 M	49.5	1115.4	346.5
BLE 5	nRF52840	32-bit ARM Cortex-M4F @ 64MHz	2 M	2.1 × 10^−3^	40.8	19.2
	CC2640R2F	32-bit ARM Cortex-M0 @ 32 MHz	2 M	9 × 10^−3^	27.3	18.3
	CC2650 RF Core	32-bit ARM Cortex-M0 @ 32 MHz	1 M	8.1 × 10^−3^	27.3	18.3
BLE 4.2	RN4871	8-bit Microchip 8051 @ 16 MHz	10 k	-	39	39
	PAN1760A	32-bit ARM Cortex-M0 @ 32 MHz	115.2 k	1.65 × 10^−4^	10.89	10.89
ZigBee	XB24-AUI-001	-	250 k	-	148.5	165
	ATSAMR21B18	32-bit ATSAMR21 ARM Cortex-M0+ @ 48 MHz	-	8.1 × 10^−3^	41.4	35.4
Z-Wave	ZM5304	8-bit Microchip 8051 @ 16 MHz	9.6/40/100k	5.61 × 10^−3^	140.91	107.91

Please refer to the datasheets for various modules.

**Table 3:** Typical microprocessor modules and parameters.

Microprocessor	Data Bus (bit)	Flash (KB)	RAM (KB)	Active Consumption (μW)	Sleep Consumption (μW)
ATmega2560	8	256	8	900	0.18 (Power down)
MSP430F5529	16	128	8	6960	6.3 (Standby)
ATSAMD21G18 @ 48MHz	32	256	32	12210	15.18 (Standby)
STM32F7xx	32	1024	64	12600	180

Please refer to the datasheets for various modules.

**Table 4:** Typical sensor modules and parameters.

Sensor Type	Sensor	Resolution	Dara Rate (Hz)	Power Consumption	
				Measurement (μW)	Standby (μW)
Accelerometer	ADXL345	10 bits	Max. 3200	100	0.25
	MPU-6050	16 bits	Max. 1000	1650	16.5
	LIS2DS12TR	16 bits	Max. 6400	270	1.26
Temperature	TMP006	0.03125 °C	1000	792	-
	D6T-44L-06	0.14 °C	-	25	-
Pressure	BMP280	0.18 Pa	1	9.042	0.33
Humidity	HDC1000	14 bits	1	2.46	1
Light	OPT3001	0.01 lux	1	4.5	-

Please refer to the datasheets for various modules.

**Table 5:** Comparison of some electromagnetic generators found in the literature.

Year	Frequency (Hz)	Acceleration (g)	Internal Resistance (Ω)	Power Density (μW/cm^3^)	Device Volume (cm^3^)	Features
2010 [135]	30	-	25	0.008	50.26	Harvest from flow-induced vibration
2013 [136]	840/1070/1490	1.0	626	0.157/0.014/0.117	0.035	With three modes and multiple frequencies; Integrated with MEMS.
2015 [137]	10	-	1.19	2187.5 max.	160	Work at low frequencies; Generate much more power than a traditional one.
2016 [138]	2 to 5	0.08 to 0.19	-	0.123	42.2	A curved electromagnetic generator with lightweight designed for wearable electronics.
2016 [139]	2.65	0.15	106	4.24 max.	29.5	Suit for low resonance-frequency vibrations; Provide sufficient energy for WSNs.
2017 [140]	8.5	0.3	800	256	97.0	Work at low frequencies; Harvest enough power for measurement and transmission the temperature and humidity every 20 s.
2018 [141]	20	-	86.9	145.08 max.	13.854	Absorb gas pressure fluctuations; With a higher power density.
2018 [142]	10 to 80	3	-	8.75	215.98	A wide bandwidth of 70 Hz; Potential for biomedical use.
2018 [143]	1	-	240	3.05 max.	20.11	With a sprung eccentric rotor; Suit for the low-frequency excitations.

**Table 6:** Comparison of various energy harvesting technologies.

Energy Sources	Source Characteristic [177]	Conversion Efficiency [177]	Harvested Power [177]	Technologies/Device	Advantages	Disadvantages
Light energy	Indoor: 0.1 mW/cm^2^	10–24%	10 μW/cm^2^	Photovoltaic EH/generators	Green, renewable, inexhaustible energy source; With relatively high conversion efficiency and low noise.	Bulky electronics, like solar panels; Affected by weather, regions, locations (inside or outside buildings); With high cost and pollution.
	Outdoor: 100 mW/cm^2^		10 mW/cm^2^			
RF energy	900 MHz: 0.3 μW/cm^2^	50%	0.1 μW/cm^2^	RF EH/generators	Green and efficient source of energy; Long effective energy transfer distance; Prolong the lifetime of electronics and WSNs.	Ultra-low output power and often varies in time due to the decrease of the circuit performance.
	1800MHz: 0.1 μW/cm^2^					
Thermal energy	Machine: 100 mW/cm^2^	3%	1–10 mW/cm^2^	Thermoelectric EH/(nano)generators	With high durability, precision, small volume; Collecting residual thermal energy; Safety and reliability.	Difficult to integrate with MEMS; With relative low conversion efficiency.
				Pyroelectric EH/(nano)generators	With relative higher conversion efficiency; Can be fabricated as micro/nanoscale.	Require high-frequency temperature fluctuations and high efficiency of energy extraction cycles.

The source characteristic, conversion efficiency and harvested power are referenced from the introduction to energy harvesting concluded by an applications engineer with the single cell battery charge management group at Texas Instruments in 2015 [177].

**Table 7:** Comparison of various energy harvesting technologies.

Energy Sources	Source Characteristic [177]	Conversion Efficiency [177]	Harvested Power [177]	Technologies/Device	Advantages	Disadvantages
Mechanical energy	Machine: 1 m @ 5 Hz 10/s^2^ @ 1 kHz	Source dependant	100 μW/cm^2^	Piezoelectric EH/(nano)generators	With higher power density, and high output voltage; With compact and simple architectures; Can be fabricated and directly integrated into MEMS; Easy to scale down to nanoscale.	Energy harvesting devices are easy to fatigue and crack; Work at a low and narrow frequency band; Limitation on the types of optional materials and complexity of fabrication techniques.
				Electromagnetic EH/generators	With high efficiency and high output current; With low cost; Easy to scale up; High output for large-scale devices.	With inevitable coil losses; Difficulty to fabricate microscale devices and integrate with MEMS; May be interfered by the electromagnetic waves.
				Triboelectric EH/(nano)generators	Simple, low weight, cost-efficient, scalable, robust and reliable; With high efficiency.	With inevitable wear of materials; Heat generated by the wear may cause catastrophic accidents.
				Electrostatic EH/(nano)generators	With high output voltage; With low cost; Easy to fabricate with MEMS and integrate with other devices.	Most require separate voltage sources or electret materials or doublers; Quite small energy density and low output; Work at a low and narrow frequency range.

The source characteristic, conversion efficiency and harvested power are referenced from the introduction to energy harvesting concluded by an applications engineer with the single cell battery charge management group at Texas Instruments in 2015 [177].

**Table 8:** Condition monitoring techniques.

Condition Monitoring Methods	Techniques
Feature extraction [189]	Time domain—mean value, variance, root mean square (RMS), kurtosis, skewness, entropy, time synchronous average (TSA) etc.; Frequency domain—fast Fourier transform (FFT), envelope, bispectrum, modulation signal bispectrum (MSB), etc.; Time-Frequency—short-time Fourier transform (STFT), Wigner-Ville distribution (WVD), wavelet, etc.; Other methods—spectral kurtosis (SK), principal component analysis (PCA), independent component analysis (ICA), empirical mode decomposition (EMD), Local mean decomposition (LMD), etc.
Modelling [190]	Numerical modelling; Data modelling.
Machine learning [191]	Neural network, deep learning, expert system, fuzzy logic, support vector machine (SVM), etc.
